# Elucidating Berberine’s Therapeutic and Photosensitizer Potential through Nanomedicine Tools

**DOI:** 10.3390/pharmaceutics15092282

**Published:** 2023-09-05

**Authors:** Célia Marques, Maria Helena Fernandes, Sofia A. Costa Lima

**Affiliations:** 1 IUCS-CESPU, University Institute of Health Sciences (IUCS), CESPU, CRL, 4585-116 Gandra, Portugal; celia.m@netcabo.pt; 2 LAQV, REQUIMTE, Faculdade de Farmácia, Universidade do Porto, 4050-313 Porto, Portugal; 3 BoneLab-Laboratory for Bone Metabolism and Regeneration, Faculty of Dental Medicine, University of Porto, LAQV, REQUIMTE, U. Porto, 4200-393 Porto, Portugal; 4 LAQV, REQUIMTE, Instituto de Ciências Biomédicas de Abel Salazar, Universidade do Porto, 4050-313 Porto, Portugal

**Keywords:** berberine, delivery systems, polymeric nanoparticles, pharmacokinetics, photodynamic therapy

## Abstract

Berberine, an isoquinoline alkaloid extracted from plants of the *Berberidaceae* family, has been gaining interest due to anti-inflammatory and antioxidant activities, as well as neuro and cardiovascular protective effects in animal models. Recently, photodynamic therapy demonstrated successful application in many fields of medicine. This innovative, non-invasive treatment modality requires a photosensitizer, light, and oxygen. In particular, the photosensitizer can selectively accumulate in diseased tissues without damaging healthy cells. Berberine’s physicochemical properties allow its use as a photosensitising agent for photodynamic therapy, enabling reactive oxygen species production and thus potentiating treatment efficacy. However, berberine exhibits poor aqueous solubility, low oral bioavailability, poor cellular permeability, and poor gastrointestinal absorption that hamper its therapeutic and photodynamic efficacy. Nanotechnology has been used to minimize berberine’s limitations with the design of drug delivery systems. Different nanoparticulate delivery systems for berberine have been used, as lipid-, inorganic- and polymeric-based nanoparticles. These berberine nanocarriers improve its therapeutic properties and photodynamic potential. More specifically, they extend its half-life, increase solubility, and allow a high permeation and targeted delivery. This review describes different nano strategies designed for berberine delivery as well as berberine’s potential as a photosensitizer for photodynamic therapy. To benefit from berberine’s overall potential, nanotechnology has been applied for berberine-mediated photodynamic therapy.

## 1. Introduction

**Figure 1:**
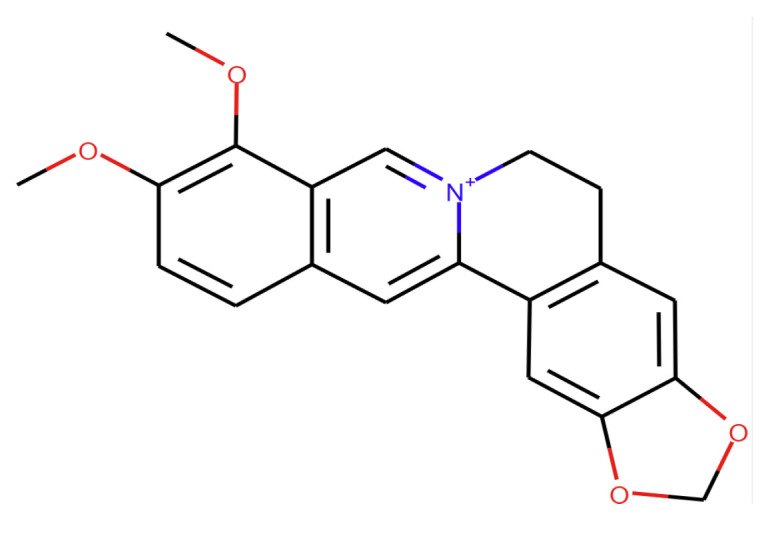
Chemical structure of berberine.

Berberine (BBR) is a common yellowish isoquinoline quaternary alkaloid (Figure 1) from the structural class of protoberberines present in the roots, rhizome, and stem bark of the plants of the *Berberidaceae* family [1].

This compound is widely used in traditional Chinese medicine to treat hypertension and inflammatory conditions, and has been attracting much attention due to its various biological activities, namely, antimalarial [2], antioxidant [3], antimicrobial [4,5], and antiviral [6]. BBR also shows antidiarrheal pharmacological properties, promotes tightness of the intestinal epithelial tight junction (TJ) barrier, ameliorates TJ barrier impairment by suppressing the production of proinflammatory cytokines [7,8,9], and can inhibit toxins and bacteria such as *Helicobacter pylori* [10]. BBR also acts on type 2 diabetes by regulating glucose metabolism through multiple mechanisms and signal pathways [11,12]. In addition, BBR has been shown to exert anticancer activity, namely, in human nasopharyngeal carcinoma [13], breast and lung cancer [14,15], human squamous carcinoma cells [16], and hepatocellular carcinoma [17]. The wide applications of BBR also include hepato-protection, and neuroprotective effects in cognitive disorders and eye diseases [18,19]. More recently, the antibacterial and bone-formation activities of BBR have been exploited in the field of dentistry [20,21]. BBR promotes osteogenic differentiation from bone marrow mesenchymal stem cells, periodontal ligament stem cells [21,22], and dental pulp stem cells through distinct activating mechanisms [23]. More recently, the potential of BBR as a photosensitizer in photodynamic therapy has also been demonstrated [24,25].

Photodynamic therapy (PDT) is an emerging non-invasive treatment strategy that can be used in the therapy of non-oncological diseases and cancer [26,27]. The possibility of being used in association with other therapeutic approaches has promoted its use in different fields of medicine, namely, oncology, dermatology, gynecology, urology, chronic inflammation, and microbial infections [28]. This technique depends on three agents: a photosensitizing chemical compound, light, and endogenous molecular oxygen. Individually, each component is non-toxic, but when a photosensitizer (PS) is irradiated with light, photochemical reactions result in the formation of highly reactive oxygen species (ROS) that are responsible for cytotoxicity and cell death [29]. The fluorescent properties of BBR are valuable for the use of PDT. Some authors have reported that BBR is a PS agent which can produce singlet oxygen and other radicals in the presence of light irradiation, potentializing treatment efficiency and minimizing the side effects [24].

Despite the proven therapeutic efficacy of BBR, some limitations constrain its pharmaceutical development, namely, poor aqueous solubility (~2.0 mg/mL), low oral bioavailability (less than 5% in plasma), and poor gastrointestinal absorption, thus being considered a class IV drug in the Biopharmaceutical Classification System [30,31,32]. To overcome the associated drawbacks of BBR, several approaches have been explored, namely, the use of permeation enhancers (e.g., spices, peptides, surfactants, and polymers) [33]. 

Nanomedicine has emerged as a tool to deliver drugs either encapsulated or functionalized on the surface to improve the therapeutic properties of drugs. The nanoparticles (NPs) used for drug delivery can be produced with different materials such as natural or synthetic polymers, lipids, metals, and others. Given its structure and the nano-sized feature, this method of delivery allows (i) protecting pharmaceutical molecules, (ii) crossing biological barriers to deliver the drug to the target place, (iii) controlling the releasing process of drugs in the body, and (iv) increasing drug circulation in the bloodstream [34,35]. In this way, the pharmacokinetic characteristics of the loaded drug are improved, resulting in better therapeutic efficacy and fewer side effects. Increasingly, nanomedicine has been applied to natural compounds [36,37]. Several nanoparticulate delivery systems for BBR have been reported. They will be revised in this work, elucidating different nano strategies designed for BBR delivery to reduce limitations and improve therapeutic efficacy as well as the photochemical characteristics and biological applications of BBR-mediated PDT.

## 2. Berberine’s Potential as a Bioactive Molecule

### 2.1. Pharmacokinetics of Berberine

**Figure 2:**
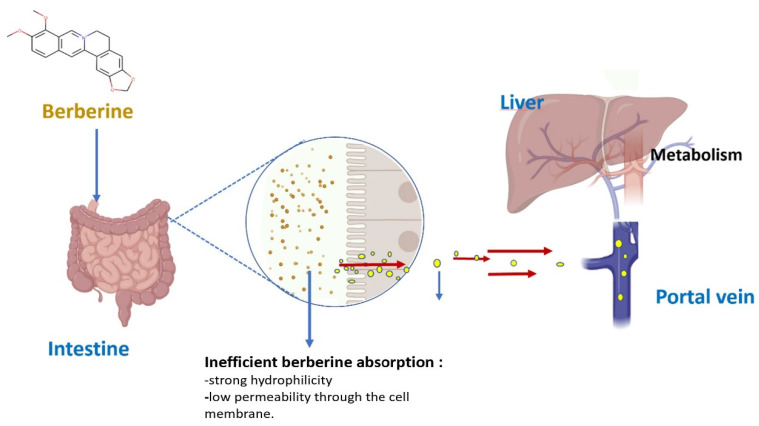
Oral administration pathways of berberine.

The main clinical BBR administration is the oral route. Upon oral administration, BBR is absorbed in the gastrointestinal tract. However, studies have shown that the intestinal absorption efficiency of BBR and its oral bioavailability are extremely low [38,39,40]. The absolute bioavailability of BBR is less than 1% after oral administration in rats, 0.68% according to Chen et al. [38], and 0.36% according to Liu et al. [40], which may be related to the compound’s physicochemical nature. Its structure (Figure 1) contains a quaternary ammonium group with strong hydrophilicity and low permeability through the cell membrane, resulting in low bioavailability [38]. This low oral bioavailability may be due to BBR’s poor absorption (56%), the first-pass effect in the intestine (43.5%), and in the liver (0.14%) [40] (Figure 2). In humans, according to Hua and co-workers, after a single oral dose of 400 mg BBR, the mean maximum plasma concentration (C_max_) in 20 volunteers was about 0.4 ng/mL, and the area under the concentration–time curves from zero to infinity (AUC0-∞) was only about 9.18 h ng/mL [41].

Several barriers have been identified that hamper BBR oral administration, namely: (i) the formation of self-aggregates in the stomach acidic environment and the small intestine; (ii) interaction with multidrug transporter P-glycoprotein [42]; and (iii) hepatobiliary re-excretion due to the existence of the hepatoenteral circulation process [32,43].

Animal studies show that after oral administration, BBR could be widely distributed in several organs, including kidneys, muscles, lungs, brain, heart, and pancreas [44,45], but predominantly in the liver, intestinal tract, kidney, and lung [43]. Moreover, BBR and its bioactive metabolite concentrations were higher in organs than in blood [44]. Overall, BBR is considered a compound with a good safety profile and a high therapeutic potential; yet some degree of toxicity, genotoxicity, mutagenicity, carcinogenicity, and cardiotoxicity has been reported in experimental models. Thus, the safety of BBR is closely related to the experimental model, the route and duration of administration, as well as the concentration/dose used [46]. In clinical studies, BBR toxicity has also been reported, namely, as gastrointestinal problems in patients with type 2 diabetes upon oral administration. The toxicity of BBR is still a poorly studied aspect and remains a controversial topic of discussion [47]. In 2013, Ma and co-workers evaluated the excretion of BBR in a study using rats, where it was administered orally at 200 mg/kg. After 48 h of administration, 22.8% of the administered dose was recovered in bile (9.2 × 10^−6^%), urine (0.094%), and feces (23%). Of the total excreted, 20% was as BBR, and 3.8% was in the form of the major metabolites [48]. Overall, BBR has poor absorption in the gut; thus, after oral administration, it remains within the gastrointestinal lumen and is finally excreted in the feces. The absorbed fraction in the gastrointestinal tract is mainly distributed through the different organs, and it has been shown that in all tissues studied its concentration is higher than in plasma within 4 h of administration [44]. Absorbed BBR could be converted into metabolites, and so far, 97 BBR-related metabolites have been identified in rats [49,50].

### 2.2. Berberine’s Metabolites and Their Pharmacological Effects

The metabolic pathways of BBR can be separated into phase I, with metabolites, namely, berberrubine, demethyleneberberine, jatrorrhizine, thalifendine, and palmatine [39], and phase II, known as conjugation reactions with glucuronic acid, sulfuric acid, or methyl groups [50]. The phase-I metabolites of BBR were mainly produced via demethylation, demethylation, reduction, and hydroxylation [49]. Demethylation is a primary metabolic product of BBR that leads to berberrubine and thalifendine as the main metabolites in rats and humans [50]. However, some metabolites of BBR that preserve the methoxyl groups (e.g., palmatine and jatrorrhizine) could be further metabolized through mono-, di- or tri-demethylation in rats [50,51]. Demethyleneberberine, another important BBR metabolite, is formed via demethylation by cleaving the dioxymethylene five-membered ring [52,53]. Hydroxylation has been reported as a central metabolic pathway for palmatine and jatrorrhizine, although studies have shown that jatrorrhizine is also metabolized via the reduction pathway just as columbamine is [54]. In the liver and the intestine, phase-I metabolism is mediated through cytochrome P450 enzymes (CYPs). Clinical studies have shown that CYP2D6 is the primary human cytochrome for producing BBR’s metabolites, followed by CYP1A2, 3A4, 2E1, and CYP2C19 [18,50]. CYP2D6 and CYP1A2 play a significant role in transforming BBR into thalifendine, and CYP2D6, CYP1A2, and CYP3A4 in demethyleneberberine production. BBR phase-I products are then conjugated with glucuronic acid or sulfuric acid to form phase-II metabolites rapidly, and are finally excreted in the urine and bile [18,48]. Glucuronidation is mediated through UDP-glucuronosyltransferases (UGTs), including UGT1, 2, 3, and 8 families [50]. Berberrubine and demethyleneberberine can be glucuronidated by UGT1A1 and UGT2B1, while the glucuronidation of demethyleneberberine is favored by UGT1A1 [53]. Many studies revealed that some of the metabolites of BBR, such as berberrubine, columbamine, and demethyleneberberine, exert a biological activity similar to that of BBR, exhibiting antioxidant, anti-inflammatory, antimicrobial, antitumor, neuroprotective, hepatoprotective, hypolipidemic, and hypoglycemic effects, which give it an essential role in pharmacological therapy of various diseases [50].

Berberrubine is the primary metabolite of 9-demethylated BBR with potential pharmacological activities, namely, the scavenging effect of ROS, hypoglycemic activity, and anti-inflammatory activity [55]. In a study by Jang et al. where an electron spin resonance spectrometry method was used, it was shown that the •OH scavenging activities of berberrubine and BBR, in a concentration of 1 mM, were 85% and 23%, respectively. These •OH scavenging activities were closely related to their ferrous ion chelating activities [56]. In addition, berberrubine also showed hypoglycemic activity, compared to the anti-diabetic effects of BBR on type 2 diabetes [11,12]. When administered orally to rats, berberrubine is rapidly metabolized into berberrubine-9-O-β-D-glucuronide. Yang and co-workers demonstrate that, in human normal liver cell line L-O_2_ in vitro, treatment with berberrubine or berberrubine-9-O-β-D-glucuronide in concentrations of 5, 20, 50 μmol/L increased glucose consumption, enhanced glycogenesis, stimulated the uptake of the glucose analog 2-NBDG, and modulated the mRNA levels of glucose-6-phosphatase and hexokinase, which means that berberrubine and berberrubine-9-O-β-D-glucuronide were potential agents for reducing glucose levels [57]. As an anti-inflammatory, berberrubine has demonstrated efficacy in the treatment of ulcerative colitis in rats; as shown in the study by Yu and co-workers, 20 mg/kg of berberrubine produced a therapeutic effect comparable to that of 50 mg/kg of BBR and that of the positive control 200 mg/kg of sulfasalazine, significantly reducing the disease activity index with a prolonged length of the colon and an increase in body weight. In addition, berberrubine markedly attenuated colonic inflammation, attenuating inflammatory cell infiltration and inhibiting the production of myeloperoxidase and cytokines (TNF-α, IFN-γ, IL-1β, IL-6, IL-4, and IL-10) in mice with the disease [58]. Berberrubine is also known to have anti-inflammatory effects via mechanisms similar to those of BBR, which possibly benefited from its inhibition of the production of IL-8 [59]. Some studies point to berberrubine’s antitumor activity as a result of the hydroxyl group in the C-9 position, as well as indicating that topoisomerase IIα was the cellular target of berberrubine, which was seen as a specific poison of DNA topoisomerase II by stabilizing the topoisomerase II-mediated cleavable complex in vitro [60,61].

Columbamine has been identified as a hydrogenation metabolite of BBR in humans and rats, resulting from the metabolic pathway of BBR in vivo reduction [52,62]. Anti-inflammation and pain relief, hypoglycemic effects, and hypolipidemic effects are some of the activities in which columbine has been shown to play an important role [63,64,65], and more recently, scientific evidence of its antitumor effects has emerged. Yang et al.’s study shows that in melanoma cells, columbamine inhibited melanoma cell proliferation, migration, and invasion in A375 cells. Phosphorylation of STAT3 and expression of HSP90 were also repressed by columbamine in a concentration-dependent manner [66]. In colon cancer, Lei and collaborators found that columbamine inhibited the proliferation, migration, and invasion process of colon cancer cells, and promoted apoptosis via suppression of the Wnt/b-catenin signaling pathway, thereby inhibiting the development of colon cancer [67]. In addition, columbamine promotes cell apoptosis, inhibits proliferation and migration, stokes growth in hepatocellular carcinoma cells, and represses cell migration through the inactivation of PI3K/AKT, p38, and ERK1/2 MAPKs signaling pathways [68].

BBR demethylation results in demethyleneberberine, a major BRR’s metabolite in vivo. Demethyleneberberine has been studied for its various pharmacological properties, ranging from its anti-inflammatory and immunoregulation effects to blocking the maturation of IL-1β in a mitochondria-dependent manner by inhibiting TLR4-mitochondria signaling [69]. The hepatoprotective and anti-fibrotic effects were also studied by Wang and co-workers, who found that demethyleneberberine protects against thioacetamide-induced hepatic fibrosis in mice reduces the expression of TGF-β1/Smad signaling and TIMPs (tissue inhibitors of matrix metalloproteinase), and exhibits a higher safety profile as compared to BBR [70]. In addition, demethyleneberberine is a natural mitochondria-targeted antioxidant that can inhibit mitochondrial dysfunction, oxidative stress, and steatosis in an alcoholic hepatic disease model [71]. Studies of the neurodegenerative and anti-tumoral activity of demethyleneberberine have also been carried out [72,73,74].

The efficient inhibitory effect on lung cancer cell proliferation and migration by inducing cell cycle arrest and triggering cellular senescence by downregulating the c-Myc/HIF-1α pathway has been noted [72]. In colon cancer, demethyleneberberine could induce apoptosis and effectively inhibit TGF-β/Smad-induced epidermal mesenchymal transition in HCT-116, exhibiting a more effective inhibition ability on the colon cancer cell proliferation than BBR [74].

### 2.3. Nanotechnology-Based Strategies for Berberine Delivery

**Figure 3:**
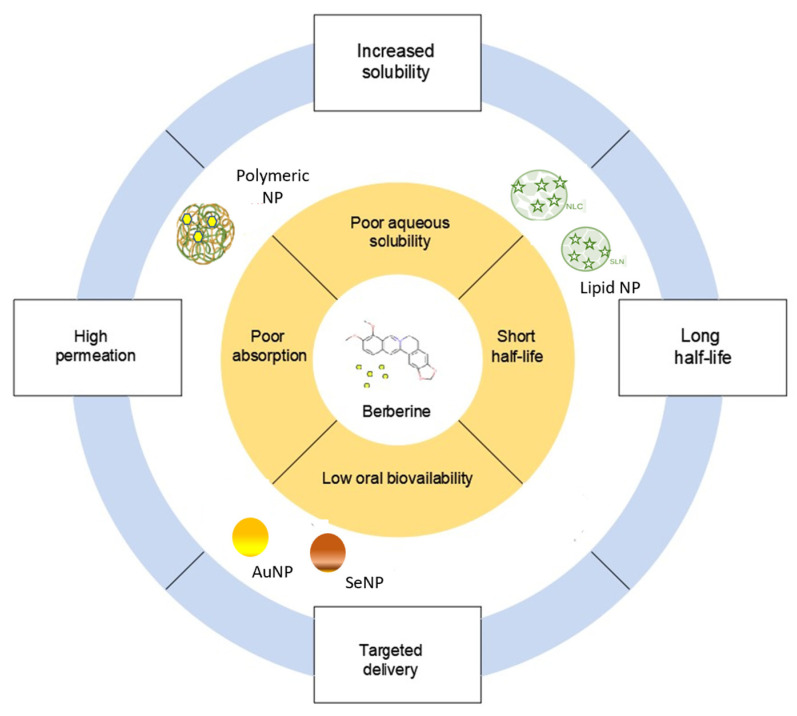
Advantages of incorporating berberine into nanocarriers as a therapeutic agent.

Nanotechnology has opened new horizons for the development of drug delivery systems to improve diagnosis and therapy in different areas of health. Nanoparticle-based delivery systems have been developed to reduce BBR oral administration limitations and thus improve BBR efficacy (Figure 3). Various types of nanocarriers (e.g., polymeric-based, lipid-based, silver, and gold nanoparticles) have been used as delivery systems of BBR [75]. The benefits of a nanomedicine tool contribute to overcoming the limitations of BBR, namely, its poor aqueous solubility, slight absorption, and low bioavailability.

#### 2.3.1. Lipid-Based Nanoparticles

Some lipid-based delivery systems for BBR have been described [76]. Recently, Raju and collaborators developed BBR-loaded nanostructured lipid carriers (NLC) with a melt-emulsification and ultrasonication method optimized by a 3^2^-factorial design for oral Alzheimer’s therapy. Optimized lipid nanoparticles (NP) presented a particle size of 186 nm, and zeta potential of −36.86 mV with 88% entrapment efficiency. Pharmacodynamic studies demonstrated improved behavioural parameters in vivo by the nanoparticle compared to pure BBR in Albino Wistar rats [77]. Lipid nanoparticles prepared with a solid lipid and a surfactant (solid lipid nanoparticles, SLNs) attenuated in vitro inflammation in rat cardiomyocytes. BBR-loaded SLNs exhibited a particle size of 13 ± 1 nm, and the zeta potential of −1.05 ± 0.08 mV with an entrapment efficiency of 50 ± 5%. Inflammation and oxidative stress markers were lower with BBR and BBR-loaded SLNs. The attenuation of ROS generation and apoptosis of cardiomyocytes suggest the potential of BBR-loaded SLNs to prevent doxorubicin-induced inflammation and oxidative stress in rat cardiomyocytes [78]. Hybrid nanoparticles combining lipid nanoparticles with polymers were designed to delivery BBR across the blood brain barrier through intranasal administration. Nanostructured lipid carriers loaded with BBR were coated with chitosan by hot homogenization and ultrasonication. The optimized formulation had a size of 181 ± 4 nm, and positive surface charge of +36.8 mV due to chitosan coating. Ex vivo permeation via nasal mucosa increased in relation to free BBR, exhibiting a safe nasal delivery. Pharmacokinetic and brain accumulation studies showed that the intranasal administration of the optimized nanocarrier had substantially greater drug levels in the brain. The ratios of BBR brain/blood levels at 30 min, AUC_brain_/AUC_blood_, drug transport percentage, and drug targeting efficiency for BBR were higher for BBR-loaded nanoparticles compared to the free BBR solution, suggesting enhanced brain targeting [79]. 

#### 2.3.2. Inorganic-Based Nanoparticles

Kim and co-workers developed organic/inorganic hybrid nanoparticles made of BBR and zinc oxide for the therapy of lung cancers. The BBR-loaded ZnO nanoparticles had a 200–300 nm hydrodynamic size and improved antiproliferation efficacy in human lung adenocarcinoma cells, based on the chemo-photothermal therapeutic efficacy. Upon intravenous administration in rats, BBR-loaded ZnO nanoparticles did not induce severe hepatotoxicity, renal toxicity, and hemotoxicity. The designed hybrid nanoparticles may represent a nanomedicine tool for efficient and safe chemo-photothermal therapy of lung cancers [80].

BBR-loaded gold nanoparticles (AuNPs) promoted apoptosis in breast cancer. Chui et al. produced AuNPs with biocompatible collagen and then conjugated them with BBR. The particle size of the nanocarrier was around 227 nm, and exhibited less cytotoxicity to bovine aortic endothelial cells vs. Her-2 cell line. The nanocarrier was internalized by clathrin-mediated endocytosis and led to cell autophagy. In an experimental model of xenograft mice treated with the nanocarrier, tumor suppression was observed [81]. 

Given the chemo-preventative and therapeutic properties of selenium nanoparticles (SeNPs), Othman et al. produced and characterized SeNPs loaded with BBR to evaluate their anticancer potential [82]. Swiss albino mice were injected with Ehrlich ascites tumor cells, and then treated with BBR-loaded SeNPs. This nanocarrier significantly improved survival rate and decreased body weight and tumor size, compared to the untreated group. BBR-loaded SeNPs reduced oxidative stress and nitric oxide levels, and increased glutathione levels. Additionally, BBR-loaded SeNPs induced an apoptotic cascade in the tumor cells. This work reveals a new insight into the potential role of green-synthesized SeNPs for chemotherapy.

#### 2.3.3. Polymeric-Based Nanoparticles

Polymeric NPs are being widely explored for the therapeutic development of novel drug delivery systems to improve drug stability, solubility, and safety [83]. So far, both natural and synthetic polymers have been used in BBR delivery. Among the natural polymers, whose origin can be animal, plant, or microbial [84], the most commonly used include alginate, dextran, chitosan, gelatin, collagen, and albumin. Synthetic polymers include poly lactic-co-glycolic acid (PLGA), polylactic acid (PLA), poly (ethylene glycol) (PEG), poly (ε-caprolactone) (PCL), polymethacrylates, and polyvinyl pyrrolidone [85].

##### Natural Polymers

The properties of natural polymers guide their application as drug delivery systems. These polymers can be found abundantly in nature; they are biocompatible and biodegradable, and have low toxicity. However, their use can be challenging due to batch-to-batch variability related to difficulties in purification and their wide molecular weight distributions [85].

Chitosan is the most extensively studied biopolymer for medical applications. It is a hydrophilic cationic polymer obtained by alkaline hydrolysis of chitin. It is biocompatible and biodegradable, making it appropriate for clinical use. Chitosan NPs can be produced using either bottom-up or top-down approaches and/or a combination of both procedures [86]. Wang and collaborators studied the effectiveness of BBR hydrochloride-loaded chitosan nanoparticles in treating nasopharyngeal carcinoma. The ionic cross-linking technique prepared folate acid-modified chitosan NPs loaded with BBR hydrochloride (BH/FA-CTS NPs). The physicochemical properties of BH/FA-CTS NPs and their effect on proliferation, migration, and apoptosis in human nasopharyngeal carcinoma cells were studied in vitro and in vivo. The in vivo study used tumor-bearing mice, whose tumors were initially allowed to reach 4–5 mm in diameter. Four groups of tumor-bearing mice were used (n = 6) and injected with BBR of 20 mg/kg as (1) BH/FA-CTS NPs; (2) BH/CTS NPs; (3) free BBR solution; and (4) 0.9% sodium chloride solution (blank control). Data demonstrated that the NPs can effectively improve BBR’s bioavailability and may potentially improve nasopharyngeal carcinoma treatment [13].

In another study, a nanocarrier based on chitosan and fucoidan was developed for oral delivery of BBR towards defective intestinal epithelial TJ barrier therapy [87]. Taurine is an inhibitor of inflammatory factors and reduces the dysfunction of epithelial cells. Fucoidan can enhance epithelial barrier function via upregulating the expression of the TJ protein Claudin-1, which causes improved epithelial barrier function. A sulfonated fucoidan, fucoidan-taurine (FD-Tau) conjugate, was self-assembled with BBR and chitosan to form BBR-loaded chitosan/FD-Tau complex NPs to be used in the treatment of defective intestinal epithelial TJ barrier caused by bacterial endotoxin, demonstrating that it has the potential to do so [87]. BBR-loaded chitosan NPs have also been effective with a favorable release profile and protective efficacy against osteoarthritis [88]. Their output showed that upon an intra-articular injection, in each of the groups with 0.6 mg/mL BBR-loaded chitosan NPs (containing 60 µg/mL BBR) or 60 µg/mL of BBR dispersed in 50 µL of PBS, NPs reduced BBR leakage in the blood and consequently led to greater retention of free BBR in the synovial fluid of the joint cavity [88]. 

In the study by Lin and co-workers, fucose-chitosan/heparin NPs loaded with BBR interacted with *Helicobacter pylori* and enhanced the suppressive effect of BBR on bacteria growth. The natural compound was protected by the fucose-conjugated NP from destruction by gastric acids, allowing extra concentration of BBR in the mucus layer, with promising potential to manage gastric inflammation [10]. Saleh and co-workers used BBR-loaded chitosan NPs to investigate the neuroprotective effects against scopolamine-induced cognitive impairments in Alzheimer’s disease. The results showed a better efficacy of drug-loaded NPs than free BBR, with a significant improvement in learning and memory function [89].

Dextran, a polysaccharide, has been employed in a range of biomedical applications as a drug carrier owing to several advantages such as a well-defined structure, high stability of glycosidic bonds, low pharmacological activity, and protection of conjugated drugs from biodegradation [90]. In vitro, O-hexadecyl-dextran NPs loaded with BBR (0.0125 µg) reduced oxidative stress in rat hepatocytes at a concentration 20 times lower than free BBR (0.25 µg), prevented high glucose stress, and improved the cytoprotective effect of the natural compound [90].

Alginate, a biopolymer usually extracted from brown seaweed, is classified as an anionic mucoadhesive hydrophilic polymer, soluble in water, forming a thick viscous, smooth gel [91]. The formulation of BBR in floating calcium alginate beads for targeting the gastric mucosa and prolonging their gastric residence time was developed by Zhang and collaborators. In vitro release studies and in vivo studies with rats verified a significant increase in the gastric residence time of beads [12]. Alginate-based composite microspheres coated with BBR were obtained with various blending ratios. Experimental data demonstrated that the formulations exhibited antibacterial activity against *Staphylococcus aureus* (*S. aureus*) and an enhanced hemostatic effect by increasing adhesion and aggregation of blood cells, giving them potential to be used as a hemostatic and to prevent infections in trauma situations [92].

BBR was incorporated within gelatin and alginate hydrogels as an antibacterial strategy against *S. aureus*. The interconnected porous network allowed an effective transport of oxygen to the environment, a sustained BBR release over 168 h, and absorption of exudates, resulting in effective accelerating wound healing [93]. Another hydrogel obtained by graft copolymerization of N-isopropyl acrylamide and alginate loaded with BBR showed a significantly improved repairing of *S. aureus*-infected wounds. This delivery system leads to granulation and capillary formation, wound closure, moisture regulation, and hemostasis, thus presenting itself as a promising wound dressing for the treatment of infected wounds or emergent trauma [9].

##### Synthetic Polymers

Synthetic polymers such as PLA, PLGA, PCL, and poly (amino acids) offer the advantage of being more reproducible for their variety of compositions with readily adjustable properties [85]. 

PLGA is a class of synthetic, biodegradable, block copolymers obtained from lactic acid and glycolic acid that have been extensively studied for drug delivery due to their biodegradability, biocompatibility, and versatile nature, and have therefore been approved by the Food and Drug Administration (FDA) [94,95]. Khan and co-workers evaluated the synergistic effect of doxorubicin and BBR as an anticancer therapy. Doxorubicin was efficiently conjugated to PLGA via carbodiimide chemistry. This conjugate was then loaded with BBR, leading to a significant alteration (depolarization) in mitochondrial membrane permeability, arrest of cell cycle progression at sub-G1 phase, and thus death in a breast cancer cell line. The overall approach confirmed the synergistic effect between doxorubicin and BBR, and reduced the toxicity associated with both compounds [96]. Hybrid NPs of PEG–lipid–PLGA were loaded with BBR using a solvent evaporation method for enhancing drug oral efficiency. The designed delivery system allowed a controlled release of BBR, and in vivo studies with equivalent doses of 50 mg/kg of BBR showed that after oral administration, intestinal uptake and bioavailability significantly increased compared to BBR alone [97].

Another hybrid NP obtained with hyaluronic acid-grafted PLGA was loaded with BBR towards Ehrlich ascites tumors, overexpressing hyaluronic acid receptors. The NPs were injected intravenously in Ehrlich ascites carcinoma (EAC)-bearing mice. BBR-loaded NPs were found to significantly enhance apoptosis, sub-G1 content, life span, mean survival time, and ROS levels in EAC cells, with a subsequent decrease in mitochondrial membrane potential and tumor burden in tumor-bearing mice [98]. BBR-loaded PLGA NPs were developed by Ahrari et al. for liver disease therapy. The delivery system prevented liver injury in rats more efficiently than free BBR, as the specific hepatic biomarkers’ expression level was strongly decreased [99].

Due to its biocompatibility and biodegradability, PLA has also been extensively used as a biomaterial in drug delivery systems. Ghaffarzadegan and co-workers prepared and optimized nano-sized core-shell NPs of BBR-loaded PLA using coaxial electrospray to solve the poor bioavailability of BBR. The NPs significantly enhanced the cytotoxicity of BBR in comparison with pure BBR against a colon cancer cell line but not in fibroblasts. Thus, the nanocarrier delivered the drug more efficiently into cancer cells [100]. In the study of Liu X et al., an amphiphilic block copolymer composed of a hydrophilic block PEG and a degradable and hydrophobic PLA was synthesized to obtain a self-assembly micelle by co-solvent evaporation and membrane hydration [101]. Cycloprotoberberine derivative (A35) and BBR were encapsulated in the micelles’ core. When compared, the A35-loaded filo micelles obtained better results than the BBR-loaded filo micelles in antitumor activity, and it was shown that cycloprotoberberine-loaded filo micelles prepared from PEG-PLA copolymers are promising for anticancer applications [101].

### 2.4. Berberine—A Tool for Photodynamic Therapy

#### 2.4.1. Principle and Main Components of a Photodynamic Therapy

Photodynamic therapy was developed in the last 100 years and has been applied in different areas of medicine such as cancer, antibacterial infections, and dentistry [29,102]. PDT initiates when a PS agent is irradiated with light at a specific wavelength; then electrons relocate to higher energy orbitals, and the PS is promoted from the ground state to the excited state. The electrons are liable to lose their excess energy and return to the ground state by emitting light, fluorescence, or heat. The photochemical reactions result in the formation of ROS, including singlet oxygen (^1^O_2_), superoxide radical anion (O_2_^•−^), hydrogen peroxide, and hydroxyl radicals (^•^OH), which are highly cytotoxic [103]. 

Two competing reactions (I and II) of the excited PS can occur [102]. In type I reactions, there is an electron exchange between the PS and the components of the system, generating radical ions that react with oxygen. The cascade of reactions initiated leads to oxidative stress. In type II reactions, energy transfer and the generation of singlet oxygen, which is highly cytotoxic, occurs from the PS [29]. 

Cell death mediated through PDT occurs by several mechanisms, namely, apoptosis, autophagy, and necrosis, depending on the type of PS, dose, and target location of the agent. Generally, apoptosis is the main type of cell death in PDT when PS is located in mitochondria. At the same time, necrosis is the major form of cell death induced by PS that localize in the plasma membrane mediated by the formation of ROS. Autophagy is induced when the PS is localized in the endoplasmic reticulum and lysosomes [24,104]. PS are substances able to absorb light at a specific wavelength, triggering photochemical or photophysical reactions. Ideally, a PS should exhibit, among others, the following properties: (i) stability at room temperature; (ii) photosensitivity only at a specific wavelength; (iii) high photochemical reactivity; (iv) maximum absorption of light at wavelengths from 600 nm to 800 nm, allowing light tissue penetration up to 2 cm; (v) absorbance of light in the range from 350 nm to 600 nm, preventing excessive photosensitivity caused by sunlight; (vi) minimal cytotoxicity in the dark; (vii) solubility in the tissues; and (vii) low cost, simple synthesis, and easy availability [105]. The selection of light source used in PDT should consider not only the PS but also the type of application, tissue, and location. For instance, for cutaneous application, a range between 600 and 800 nm should be considered, while for dentistry application, lower wavelengths which are less tissue-penetrating can be used [103]. In superficial diseases (e.g., oral cavity) non-coherent light sources can be used, having the advantage that various types of PS can be used for their broad emission range, and commercial and cost-effective availability [106]. The other crucial elements of PDT are the presence of oxygen and the type of PS used. These compounds must generally meet specific requirements such as (i) be a single pure compound, (ii) have a strong absorption peak in the red to near-infrared spectral region, (iii) have no dark toxicity and relatively rapid clearance from normal tissues, and (iv) should lead to a good production of ROS after irradiation [107]. PS used in PDT are included in four groups depending on their structure and origin: synthetic dyes, tetra-pyrrole structures, natural PS, and nanostructures [103,107]. Cheng and collaborators demonstrated that the fluorescent properties of BBR allow its application as a new type of PDT agent. It has been shown that BBR is a PS able to generate ROS and other radicals upon light exposure [108].

#### 2.4.2. Berberine as a Photosensitizing Agent

Berberine, like many natural products obtained from plants, holds a photoactive potential [109]. Studies have shown that BBR has photosensitive characteristics [110]. The excited triplet state of BBR with a high triplet energy (88–287 kJ/mol) and high quantum yield (0.08) is a good energy donor capable of sensitizing most biological macromolecules [111]. In addition, BBR exhibits high affinity with low-density lipoproteins (LDL) [112], which enhances its cellular uptake and allows specific targeting of tumor cells. Thus, several researchers have focused on the BBR’s potential as a PS applied in PDT for cancer therapy.

The absorbance spectrum of BBR exhibits two strong absorption peaks at wavelengths around 350 nm and 430 nm, which may vary in the literature according to the medium polarity. To use BBR as a PS, an excitation source light should be selected between 420 nm and 450 nm. Yet, for clinical application, a light with a wavelength of 450 nm has limited tissue penetration, and in the case of cutaneous administration, melanin and its antioxidant effect reduce the efficacy of PDT [113]. To benefit from BBR properties an advanced formulation is needed either with a drug combination or with a delivery system. Wang and collaborators have demonstrated that BBR-mediated PDT enhances the therapeutic effect of cisplatin towards cisplatin-resistant melanoma cancer cells through activation of a ROS/p38/caspase cascade [114]. This combined treatment, using a 420 nm-excitation light source, shows potential to be developed as a therapeutic anti-tumor drug in the future. 

In the study by Arnason and collaborators [115], where 1270 nm near-infrared phosphorescence produced by BBR was detected in mosquito larvae with near UV exposure, the phosphorescence at 1270 nm was considered direct evidence of ^1^O_2_ generation [116]. Some studies demonstrated that both type I and type II reactions participated in the photodynamic progression of BBR derivatives [108,111]. In addition, the photochemical characteristics of BBR derivatives were affected by the polarity, pH, and O_2_ content of solvents, the DNA binding, and the photochemical functional group of BBR [108,111,117]. BBR is a weak PS in water, but in non-polar environments, it can produce both ^1^O_2_ and radical species. It is proven that BBR is more prone to photochemical reactions in non-polar solvents than in polar solvents; also, the ROS yield is higher in non-polar solvents [108,118], and the quantum yield of BBR-sensitized oxidation increases with an increase in pH [119]. Other reactions upon laser irradiation of BBR exist besides photooxidation processes, including photoionization and photoexcitation of BBR in N_2_ and O_2_-saturated systems [108,111]. In addition to this, BBR also exerts an influence on DNA, whose binding increases the lifespan of the photoexcited BBR state and the generation of singlet oxygen.

In the study by Hirakawa and co-workers, BBR easily binds to DNA, forming a stable fluorescent complex of photoexcited BBR that leads to DNA cleavage. Upon DNA binding, BBR induces guanine-specific photooxidation via ^1^O_2_ formation [120]. Guanine is the most easily oxidized base of DNA [121]. Cheng et al. also demonstrated the capability of BBR to photosensitize DNA cleavage and, in a neutral aqueous solution, demonstrated that BBR reacts with hydrated electron and hydroxyl radical, forming the radical anion and neutral radical of BBR, which itself could react with guanine mononucleotide to give the guanine neutral radical. Single- and double-strand DNA at guanine moieties can be selectively cleaved by photoexcited BBR. Likewise, this study clarified the molecular mechanisms of BBR-induced photodamage of dGMP and DNA [122].

The functional groups of BBR are also crucial for its photochemical properties, particularly the methylenedioxy group at the C-2 and C-3 positions [117]. A comparison of the photoexcited oxygen activation of BBR and its derivatives, palmatine and jatrorrhizine, was carried out by Brezová and co-workers. When the methylenedioxy group at the C-2 and C-3 positions, which is photolabile, is replaced by two methoxy groups (palmatine) or C-2 methoxyl and C-3 hydroxyl (jatrorrhizine), the photochemical generation of O_2_^•−^ and ^1^O_2_ significantly decreases in the 5,5-dimethyl-1-pyrroline N-oxide solution [117]. These results elucidate that changing the substituents of the photochemical functional groups in BBR can affect the photochemical properties, providing the basis for the molecular design of BBR as PS.

#### 2.4.3. Biological Applications for Berberine-Mediated Photodynamic Therapy

With the discovery of the photochemical characteristics of BBR, the attention of scientific research has been attracted by the development of new treatments using BBR-mediated PDT. As already mentioned, BBR exhibits several biological activities, but its application in cancer has gained more popularity.

Oliveira and collaborators evaluated the use of BBR as a PS in PDT and observed the effects produced by this association in cervical carcinoma cells and immortalized keratinocytes. The radiation source used was a high-potency LED operating at 447 nm and 80 J/cm^2^ of fluency, set at 4 min/application, and the cells were treated with 2.5 µM BBR. Experimental data showed that BBR was internalized by the cells and remained for the next 48 h in the intracellular environment, preferentially in the cytoplasm. Upon application of PDT, a phototoxic effect was observed, resulting in 20% cell viability for cervical carcinoma cells and 47% cell viability for keratinocytes, suggesting low toxicity and efficient internalization. In addition, an increase in the production of ROS and caspase-3 activity was observed, indicating a preferential caspase-dependent apoptosis cell death mechanism [25].

BBR-mediated PDT was also evaluated in renal cancer therapy. In the in vitro study, cells were treated with 20 µM of BBR. A high phototoxicity effect with less than 20% of viable cells, an increase in ROS levels accompanied by an increase in autophagy levels, and apoptosis by caspase-3 activity, suggesting cell death by both mechanisms with laser irradiation at 447 nm and 80 J/cm^2^ of fluency, set at 4 min/application, were observed [24]. 

Malignant melanoma is a critical and aggressive skin tumor, considered the most severe and fatal skin cancer, with a steeply rising incidence and a less favorable prognosis due to the lack of efficient treatment. Human malignant melanoma cells were exposed to BBR-mediated PDT, BBR alone, and PDT alone. The light irradiation used was a laser source with 375 nm and the exposure was 7.2 J/cm^2^. BBR-mediated PDT induced apoptosis by upregulating a cleaved caspase-3 protein expression, the LC3-related autophagy level was upregulated, and BBR-mediated PDT activated endoplasmic reticulum stress, involving a dramatic increase in ROS. This study highlights the role of BBR in the phototoxicity of melanoma cancer cells [123].

As BBR already demonstrated a neuroprotective effect [19], and PDT is a non-invasive treatment modality, Carried and co-workers investigated the potential of BBR (200 µg/mL) as a PS and cytotoxic agent coupled with PDT in a human astrocytoma cell line. The radiation used was a blue LED source operating at 447 nm and 1.2 mW/cm^2^ of intensity, set for 4 min/application. The results indicated that BBR and PDT combined gave rise to a potent activation of the apoptosis pathway through a massive ROS production, a great extent of mitochondria depolarization, and the subsequent activation of caspases [124].

In tumor cells, the increase in cholesterol catabolism results in an over-expression of LDL receptors (B/E receptors). Hence, for different lipophilic and amphiphilic photosensitizers, LDL can act as a target in nanocarriers, enhancing these agents’ pharmacological efficacy [125]. In this context, LDL plays a vital role in delivering the PS in tumor cells. Taking advantage of these data, Andreazza et al. correlated the physicochemical parameters of the BBR association to LDL with the influence of LDL delivery on its accumulation in a glioma cell line and on its photoinduction by PDT [112]. The results obtained showed that the BBR’s association with LDL does not affect their recognition by the specific B/E receptors, and the photosensitizing potential of LDL-associated BBR increases its photocytotoxicity effects on the glioma cells [112].

#### 2.4.4. Nanomedicine-Based Tools for the Application of Berberine-Mediated Photodynamic Therapy

To develop effective PDT protocols, it is crucial to establish the selection criteria of a PS agent as well as improving its cellular uptake. As previously described, BBR has demonstrated such a wide in vitro efficacy that combined with PDT it emerges as an attractive tool towards cancer. Given the BBR’s reported low solubility and its poor permeability within cells, the development of nanoparticle-based formulations loaded with BBR to improve its effectiveness in PDT applications is of great interest. So far, only a couple of studies have applied this concept.

In cervical carcinoma cells, Floriano et al. analyzed the efficiency of BBR-containing nanoemulsions as PS agents in PDT [126]. The BBR nanoemulsion presented an average size around 220 nm, and an average zeta potential of −64 mV. A laser, during four minutes of application, operating at 447 nm and 80 J/cm^2^ of fluence, was used for irradiation. The results of this study showed that the main pathway of cell death was BBR-induced autophagy. For this experimental model, PDT in association with BBR-containing nanoemulsions increased cytotoxic potential and ROS generation, and therefore represents a promising nanomedicine tool in the treatment of cervical carcinoma mediated by PDT.

Recently, Comincini and collaborators developed BBR-loaded nanoparticles for astrocytoma cells towards a PDT stimulation [127]. In the study, two BBR salts were loaded in PLGA NPs and then coated with chitosan for further functionalization with folic acid. The nanoparticles presented a size around 200 nm and were positively charged, ca. +40 mV without folic acid and +10 mV with folic acid. These nanoparticles were effectively internalized by glioblastoma cells due to the functionalized ligand, with increased localization within the mitochondria. PDT induced with irradiation at 447 nm enhanced the viability reduction of the BBR-loaded nanoparticles with no significant cytotoxicity on healthy cells. The success of this strategy was confirmed by a significant increase in apoptosis and depolarization of mitochondria, mostly after PDT application. This study strongly supports the efficacy of BBR-loaded PLGA NPs combined with PDT as a nanomedicine tool for the treatment of glioblastoma.

A different approach was followed by Li et al., which synthesized three new BBR derivatives, and identified the ones with remarkable aggregation-induced emission properties, that display a higher singlet oxygen production ability than BBR [128]. To improve its cellular and targeted delivery, gold nanostars were produced loaded with the BBR derivative and coated with hyaluronic acid. The designed nanosystem exhibited a remarkable therapeutic effect on breast cancer by combining PDT with photothermal therapy (PTT) from the gold nanostars and the CD44-targeting capability of hyaluronic acid. The association of PDT with PTT synergistically enhanced cancer cell apoptosis/necrosis in vitro and anti-breast cancer activity in vivo. A new concept for PDT using natural product derivatives and their combination with PTT for efficient treatment of tumors was reported.

In some cases, PDT efficacy is hampered by an extreme hypoxic tumor microenvironment leading to restricted ROS production. To overcome this condition, Zheng and colleagues have designed and produced BBR-containing liposome with a near-infrared photodynamic dye IR168 [129]. Given the presence of a modified phospholipid for tumor-targeting, BBR accumulates in the tumor microenvironment and reverses hypoxia via metabolic regulation, resulting in an increased ROS. This work proposes a nanomedicine tool to overcome PDT immunotherapy resistance, strengthening BBR’s application in cancer therapy.

## 3. Conclusions

Recently, society’s interest in natural products has guided therapeutic solutions and motivated researchers toward treatments based on naturally occurring molecules. Traditional plants, including BBR, are described as exerting activity on diverse pathologies (e.g., infections and cancer). Yet, physicochemical properties hamper a successful administration of berberine, often resulting in low bioavailability and lack of efficacy. To overcome such limitations, nanosystems can be designed. This literature review describes several examples of delivery systems based on natural and synthetic polymers, which, when used, are advantageous to the therapeutic efficacy of BBR in different diseases. Combined therapeutic approaches based on PDT are gaining wide acceptance in the scientific community given PDT’s feasibility, non-invasiveness, and ease of application. So far, several research studies support that BBR has developmental potential as a PS for PDT. BBR’s extensive pharmacological activities, including its antitumor effect, offer a great advantage over other PS; yet they are hampered by the need to irradiate at lower wavelengths (<500 nm). BBR’s limitations, such as its poor stability and bioavailability, along with its high toxicity, can be overcome by its incorporation in drug delivery systems. These may also contribute to adjusting the polarity, pH, and O_2_ content that influence the photothermic characteristics of BBR. Studies of PDT mediated by BBR have proven effective, including in cancer treatments. Other health areas such as dermatology, ophthalmology, and dentistry would also benefit from this synergistic approach, given the PS characteristics of BBR. However, BBR-mediated PDT in delivery systems is scarce and still needs investigation to identify its expected health effects and future clinical applications.

## Data Availability

Not applicable.
